# Correlates of facility-based delivery among women of reproductive age from the Digo community residing in Kwale, Kenya

**DOI:** 10.1186/s13104-018-3818-3

**Published:** 2018-10-10

**Authors:** Vernon Mochache, Amyn Lakhani, Hajara El-Busaidy, Marleen Temmerman, Peter Gichangi

**Affiliations:** 1 International Centre for Reproductive Health, P.O. Box 91109-80103, Mombasa, Kenya; 2 University of Ghent, Ghent, Belgium; 3 Community Health Department, Aga Khan University, Mombasa, Kenya; 4 Department of Health, County Government of Kwale, Kwale, Kenya; 5 University of Nairobi, Nairobi, Kenya

**Keywords:** Correlates, Facility-based delivery, Women of reproductive age, Digo, Kwale, Kenya

## Abstract

**Objective:**

This study sought to describe factors associated with facility-based delivery among women of reproductive age in Kwale County, Kenya.

**Results:**

Between March and December 2015, 745 women from 15 villages were interviewed through a cross-sectional household survey. Respondents were selected using stratified, systematic sampling and completed a sexual and reproductive health questionnaire. Of 632 (85%) women who had a previous birth, 619 (98%) reported antenatal care attendance. Of these, 491 (79%) subsequently had a facility-based delivery. Factors associated with increased likelihood of facility delivery included respondent’s education (odds ratio, OR = 2.0, 95% confidence interval, CI 1.2–3.2, P = 0.004), ideal antenatal care attendance (OR = 2.3, 95% CI 1.4–3.7, P = 0.001) and pregnancy intention (OR = 1.5, 95% CI 1.0–2.2, P = 0.040). Being in a polygamous relationship (OR = 0.6, 95% CI 0.3–0.9, P = 0.024) and having a husband ≥ 40 years (OR = 0.5, 95% CI 0.3–0.9, P = 0.013) were associated with reduced likelihood of facility delivery. Respondent’s education (aOR = 1.9, 95% CI 1.1–3.3, P = 0.030) as well as ideal ANC attendance (aOR = 2.0, 95% CI 1.0–3.8, P = 0.040) remained significantly associated with facility delivery in multivariate analyses.

**Electronic supplementary material:**

The online version of this article (10.1186/s13104-018-3818-3) contains supplementary material, which is available to authorized users.

## Introduction

While obstetric delivery in a health facility has been associated with favourable maternal and neonatal outcomes, rates of facility-based delivery in the developing world remain unexpectedly low [1, 2]. This is attributed to various factors including the influence of sociocultural context, low perception of risks associated with pregnancy and childbirth, fear of discrimination during delivery as well as barriers associated with physical distance to a health facility and costs associated with delivery [3].

Two European Commission-funded projects seeking to improve uptake and utilization of maternal and child health (MCH) services, especially facility-based delivery and contraception, were implemented in Kwale county, Kenya. The Missed Opportunities in Maternal and Infant (MOMI) health project leveraged missed opportunities in the postpartum period using facility and community-based interventions [4]. The *Mama na Mtoto* (MNM) II project aimed to create demand for MCH services by enhancing community structures and meaningfully involving target communities [5].

Previous findings from these studies have shown high levels of contraceptive utilization among women of reproductive age (WRA) in this setting. This was associated with educational attainment, parity, antenatal (ANC) attendance at last delivery as well as intention to delay or stop future births [6]. Additionally, it was found that lay community health volunteers were instrumental in building demand for MCH services by acting as a bridge between the health system and surrounding communities [7].

The present study sought to further build on these findings by describing factors associated with facility-based delivery. As previously noted, while high rates of ANC attendance have been reported in this setting, the proportion of those pregnancies that are ultimately delivered at home still remains high [8, 9]. Findings from this study will inform priorities for better MCH programming and contribute to improved uptake and utilization of skilled assistance at birth.

## Main text

### Study design

Data for this study were collected through a cross-sectional household survey conducted in Kwale between March and December 2015. The methods have been comprehensively described previously [6]. Briefly, a structured sexual and reproductive health (SRH) household questionnaire (see Additional file 1) was administered to women between 18 and 45 years. A sample size of ~ 350 respondents was estimated based on an anticipated 50% frequency of facility-based deliveries in this setting, a sample design effect of 2.5, *Z*-statistic of 1.96 for a 95% confidence level in the estimation, 10% non-response rate, and a 2.5% margin of error. Stratified, systematic random sampling was used to select respondents. Every qualifying female respondent per household was included in the study unless they failed to provide written informed consent or were not resident in the village for more than 6 months.

### Data management and statistical analyses

Data were entered into a Microsoft Access (Microsoft Inc. Seattle, WA, USA) spreadsheet and migrated to Stata version 12 (StataCorp Inc., College Station, TX, USA) for statistical analyses. Demographic characteristics were summarized as counts (N)/percentages (%) for categorical data and median (IQR) for continuous data and compared using Pearson’s Chi squared test (categorical) and Wilcoxon rank-sum test (continuous).

The outcome of interest was self-reported delivery at a health facility among women reporting a previous birth. Odds of facility-based delivery were calculated among women with each characteristic of interest versus the reference group using multivariate logistic regression models with adjusted Odds Ratios (ORs) and 95% confidence intervals (CIs) being reported. All statistical tests were evaluated using a 2-sided α-value of 0.05.

### Ethical consideration

Ethical approval for the study was obtained from the Research Ethics Committee of the Aga Khan University, Nairobi (2014/REC-51) and the Ethics Review Committee of the University of Nairobi and Kenyatta National Hospital (P502/08/2014). A research permit from the National Commission for Science, Technology and Innovation (#4703) was also obtained to facilitate the conduct of research activities in the community. All participants provided written informed consent.

### Results

Between March and December 2015, a total of 745 female respondents were interviewed in 15 villages of Matuga sub-county, Kwale. Their median (IQR) age was 29 (23–37). Five hundred and sixty-eight (76%) were currently in a marital union with the median (IQR) age of their husbands/partners being 39 (30–46). Further, 646 (87%) women reported that they had ever attended school with the median (IQR) years of education being 8 (7–11). The median (IQR) ages at sexual debut and marriage were 18 (16–20) and 20 (18–23), respectively (Table 1).

**Table 1:** Demographic characteristics of household survey respondents (n = 745)

Characteristic	N (%)/median (IQR)
Respondent’s age	29 (23–37)
Husband/partner’s age	39 (30–46)
Age at sexual debut	18 (16–20)
Age at marriage/union	20 (18–23)
Marital status	
Currently married	426 (57%)
Currently living as if married	142 (19%)
Currently not in a union	177 (24%)
Ever attended school	646 (87%)
Years of education	8 (7–11)
Ever given birth	632 (85%)
Total number of births	4 (2–5)
Currently pregnant	75 (10%)
Duration (months) of current pregnancy	6 (4–7)

Six hundred and thirty-two (85%) respondents reported that they had ever given birth with the median (IQR) number of total births reported being 4 (2–5). Of these, 619 (98%) had attended ANC during their last pregnancy. The median (IQR) duration of pregnancy at the time of attending the first ANC visit was 5 (4–6) months while the median (IQR) number of ANC visits attended was 4 (3–5). Further, 75 (10%) women were currently pregnant at the time of the interview, with a median (IQR) pregnancy duration of 6 (4–7) months. Of these, 60 (81%) reported having ever attended ANC.

**Fig. 1:**
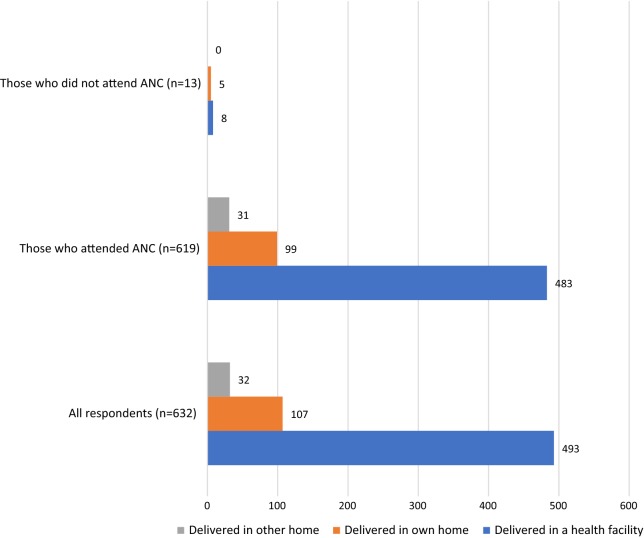
Respondents place of delivery according to status of ANC attendance

Among women who reported a previous birth, 493 (78%) had delivered in a health facility during their last pregnancy, 107 (17%) had delivered in their own home while 32 (5%) had delivered in someone else’s home. This pattern was replicated among those who had attended ANC during their last pregnancy with 483 (78%), 99 (16%) and 31 (5%) women having delivered in a health facility, in their own home and in another person’s home, respectively. Among 13 women who had not attended ANC during their last pregnancy, only 8 (60%) had delivered in a health facility (Fig. 1).

In univariate analysis, factors associated with delivery in a health facility included respondent’s education (OR = 2.0, 95% CI 1.2–3.2, P = 0.004), ideal ANC attendance (OR = 2.3, 95% CI 1.4–3.7, P = 0.001) (defined as having attended ≥ 4 ANC visits, having received information on pregnancy danger signs, having received ≥ 3 tetanus toxoid vaccinations, having received anti-malarial, anti-helminthic and hematinic supplementation), as well as pregnancy intention (OR = 1.5, 95% CI 1.0–2.2, P = 0.040). Being in a polygamous relationship (OR = 0.6, 95% CI 0.3–0.9, P = 0.024) as well as having a husband/partner who was ≥ 40 years (OR = 0.5, 95% CI 0.3–0.9, P = 0.013) were associated with reduced likelihood of a facility-based delivery.

When adjusted for age, education, marital status, ideal ANC attendance, being in a polygamous relationship and pregnancy intention; respondent’s education (aOR = 1.9, 95% CI 1.1–3.3, P = 0.030), and ideal ANC attendance (aOR = 2.0, 95% CI: 1.0–3.8, P = 0.040) remained significantly associated with facility-based delivery (Table 2).

**Table 2:** Factors associated with facility-based delivery among women of reproductive age from the Digo community residing in Kwale, Kenya (restricting to only those who reported a previous birth, n = 632)

	Univariate		Multivariate^a^	
	OR (95% CI)	P-value	OR (95% CI)	P-value
Respondent’s age (years)				
< 30	Reference			
≥ 30	0.8 (0.5–1.2)	0.217		
Husband/partner’s age (years)				
< 40	Reference			
≥ 40	0.5 (0.3–0.9)	0.013	0.7 (0.4–1.3)	0.262
Age started living with husband/partner (years)				
< 20	Reference			
≥ 20	1.1 (0.7–2.4)	0.360		
School attendance (respondent)				
Never attended school	Reference			
Ever attended school	2.0 (1.2–3.2)	0.004	1.9 (1.1–3.3)	0.030
Years of education (respondent)				
< 8 years	Reference			
≥ 8 years	1.4 (1.0–2.1)	0.072		
School attendance (husband/partner)				
Never attended school	Reference			
Ever attended school	1.3 (0.7–2.4)	0.360		
ANC attendance				
Did not attend ANC	Reference			
Attended ANC	2.4 (0.7–8.7)	0.175		
Ideal ANC attendance^b^				
No	Reference			
Yes	2.3 (1.4–3.7)	0.001	2.0 (1.0–3.8)	0.040
Marital status				
Not in a marital union	Reference			
In a marital union	1.4 (0.9–2.4)	0.159		
Not living with marital partner	Reference			
Living with marital partner	1.3 (0.8–2.1)	0.376		
Monogamous	Reference			
Polygamous	0.6 (0.3–0.9)	0.024	0.7 (0.4–1.3)	0.228
Pregnancy intention				
Didn’t want to get pregnant	Reference			
Wanted to get pregnant	1.5 (1.0–2.2)	0.040	1.5 (0.9–2.6)	0.106
Gainfully employed past 12 months				
No	Reference			
Yes	0.6 (0.4–1.2)	0.138		

^a^Adjusted for husband/partner’s age, education (both respondent’s and husband/partner’s), ideal ANC attendance, being in a polygamous relationship and pregnancy intention

^b^Constitutes having attended ≥ 4 ANC visits, having received information on pregnancy danger signs, having received ≥ 3 tetanus toxoid vaccinations, having received anti-malarial, anti-helminthic and hematinic supplementation

### Discussion

This cross-sectional survey of WRA from the Digo community resident in Kwale County, Kenya revealed a high proportion of facility-based delivery at recent birth. Women were more likely to have delivered in a health facility if they had ever attended school and if they had intended to get pregnant. Women who reported receiving ideal ANC services during their previous pregnancy were also more likely to deliver in a facility. Conversely, women were less likely to deliver in a health facility if they were in a polygamous relationship and if their husband/partner was older.

Our findings are consistent with key characteristic of pregnancy and childbirth in the developing world where the proportion of facility-based deliveries lags behind high rates of ANC attendance [10–12]. This is despite the fact that facility-based delivery has been associated with favourable maternal and neonatal outcomes. Different studies have revealed various barriers associated with this disparity including traditional and familial influences, high costs, low perceived quality of care and fear of discrimination during facility-based delivery [3, 13–16]. Women who have otherwise attended ANC well have reported fearing poor quality of services during childbirth which makes them prefer a home delivery [17–19].

Further, our findings show that the proportion of facility-based deliveries has increased over time, which is consistent with several other studies in this setting [20, 21]. This is likely related to the government of Kenya’s policy on free maternity services that was rolled out in June 2013. This policy has been credited with increasing the absolute numbers of pregnant women who deliver in health facilities [22]. However, our study showed a disparity between ANC attendance and facility-based delivery. The number of women who ultimately delivered in a health facility was lower than those who reported attending ANC in the same pregnancy.

The antenatal period offers a unique opportunity to promote healthy behaviors and practices. It serves as an ideal point during a woman’s pregnancy for discussing the choice of place of delivery [23, 24]. It also offers an opportunity to influence the decision-making process and respond to complaints and concerns [25, 26]. Our findings show that the likelihood of a facility-based delivery in this setting was more than twice as likely if a woman had received ideal ANC services during her pregnancy. In order to improve maternal and neonatal outcomes therefore, it is important to leverage demand-side factors that harness missed opportunities during ANC so as to promote uptake of facility-based delivery.

Studies have shown that educated girls and women are able to make better-informed health-related decisions [27, 28]. Successful completion of primary education among African girls has also been strongly associated with better SRH outcomes [29]. Our findings show that women who had ever attended school in this setting were twice as likely to have delivered in a health facility compared to those who had never attended school. Programs targeting to improve MCH outcomes should incorporate interventions encouraging educational attainment as well as promoting adult literacy training.

Finally, we have previously shown that pregnancy intention was associated with uptake of contraceptive services. The current findings reinforce the fact that women who plan if and when to get pregnant are more likely to deliver in a health facility. Previous studies have had mixed results regarding the role of pregnancy intendedness on influencing skilled attendance at birth especially after accounting for socioeconomic and demographic factors [30]. What is clear however, is that pregnancy intendedness could influence the quality of ANC services sought for. As our findings reveal, this could ultimately affect choice of place of delivery.

## Limitations

These findings need to be interpreted within the context of several limitations:

- Lack of data on some known correlates of facility delivery including household income as well as distance to health facility/time required to reach health facility.
- Cross-sectional study design leading to inability to infer causality.
- Self-reported responses may be subject to reporting bias.

## Additional file

**Additional file 1.** Sexual and reproductive health questionnaire. Questionnaire administered to all respondents within their households.

## Abbreviations

ANC: antenatal care
CI: confidence interval
IQR: interquartile range
MCH: maternal child health
MNM: *Mama Na Mtoto* Project
MOMI: *Missed Opportunities in Maternal and Infant Health* Project
OR: odds ratio
SRH: sexual and reproductive health
WRA: women of reproductive age
